# Imputation models and error analysis for phase contrast MR cerebral blood flow measurements in heterogeneous pediatric and adult populations

**DOI:** 10.3389/fphys.2025.1527093

**Published:** 2025-06-11

**Authors:** Eamon K. Doyle, Isabel Torres, Joseph Liu, Abhishek Karnwal, Sudarshan Ranganathan, Bradley J. De Souza, Payal Shah, Bradley S. Peterson, John C. Wood, Matthew Thomas Borzage

**Affiliations:** ^1^ Department of Radiology, Children’s Hospital Los Angeles, University of Southern California, Los Angeles, CA, United States; ^2^ Department of Pediatrics, Keck School of Medicine, University of Southern California, Los Angeles, CA, United States; ^3^ Rudi Schulte Research Institute, Santa Barbara, CA, United States; ^4^ Fetal and Neonatal Institute, Division of Neonatology, Children’s Hospital Los Angeles, Los Angeles, CA, United States; ^5^ Department of Anesthesia Critical Care Medicine, Children’s Hospital Los Angeles, Los Angeles, CA, United States; ^6^ Division of Cardiology, Department of Pediatrics, Children’s Hospital Los Angeles, Los Angeles, CA, United States; ^7^ Department of Psychiatry, Keck School of Medicine at the University of Southern California, Los Angeles, CA, United States; ^8^ Alfred E. Mann Department of Biomedical Engineering, Viterbi School of Engineering, University of Southern California, Los Angeles, CA, United States; ^9^ Department of Regulatory and Quality Sciences, Alfred E. Mann School of Pharmacy and Pharmaceutical Sciences, University of Southern California, Los Angeles, CA, United States

**Keywords:** internal carotid artery (ICA), cerebral blood flow (CBF), magnetic resonance imaging (MRI), vertebral artery (VA), phase contrast (PC)

## Abstract

Cerebral blood flow (CBF) supports brain function and health. Cerebral blood flow is affected by normal brain development, disease, medications use, and other interventions. One method to measure CBF is phase contrast magnetic resonance (PC MR) imaging, a particularly fast and reliable method to measure blood flow through major arteries such as the internal carotid (ICA) or vertebral arteries (VA). Unfortunately, sometimes PC MR can be compromised due to errors by the technologist during image acquisition, patient movement, or complex vessel structures. Our goal was to develop mathematical models to estimate CBF for a wide age range of patients whenever 1 or more vessels are not correctly measured. To investigate this, we studied a set of 258 PC MR acquisitions from a group of 196 patients with one or three acquisitions per subject (165 single images, 31 acquisitions of 3 images) ranging in age from 0.4 to 61.3 years (mean [μ] = 13.1, standard deviation [σ] = 12.3). We deliberately excluded measurements from one or more arteries in each volunteer to mimic situations with low image quality. Subsequently, we developed mathematical models to predict the missing data. Our predictive models performed well; across the human lifespan when at least one ICA measurement was available, our normalized root mean squared error values were low (<0.137), our R-squared values were high (>0.91), and we observed high intra-class correlation coefficients (>0.951). In summary, these imputation models are effective in estimating CBF in children and adults.

## 1 Introduction

Maintaining an adequate blood supply to meet the brain’s energy demand is essential for its health. The brain consumes 20% of the body’s total energy, necessitating a considerable supply of blood which constitutes 12% of the heart’s total cardiac output (Hall et al., 2012; Mink et al., 1981). Both total cerebral blood flow (CBF [mL/min]) and relative CBF adjusted by brain volume [rCBF, mL/min/100 g] also change across the lifespan, and can become impaired by injury or disease (Liu et al., 2019; Paniukov et al., 2020; Oshima et al., 2002). In children, CBF is influenced by normal brain maturation and pathological conditions such as congenital heart defects (De Silvestro et al., 2023; Lim et al., 2016), meningitis (Ashwal et al., 1992), Moyamoya (Sun et al., 2018), metabolic disorders (Qi et al., 2023), and hydrocephalous (Hirabuki et al., 2000). In adults, alterations in CBF are associated with cardiovascular risk (Hirabuki et al., 2000; Jennings et al., 2013; King et al., 2018; Varela et al., 2012), small vessel disease including Alzheimer’s dementia (Yu et al., 2020; Leijenaar et al., 2017), white matter lesions (Hanaoka et al., 2016), anemia (Borzage et al., 2016), stroke risk in sickle cell disease (Prohovnik et al., 2009; Vernooij et al., 2008), and higher risk of non-cardiovascular mortality in the elderly (Sabayan et al., 2013).

Non-invasive assessment of CBF is attractive for diagnosing and monitoring disease status. Phase contrast (PC) magnetic resonance (MR) imaging can measure flow through the internal carotid arteries (ICA) and vertebral arteries (VA). PC MR imaging provides a reproducible (intra-class correlation coefficient (ICC) 0.97–0.99, coefficient of variation 4%–9%), robust measurement of CBF that can be normalized to estimate rCBF [mL/min/100 g] (Liu et al., 2014; Sakhare et al., 2019; Koerte et al., 2013; MacDonald and Frayne, 2015). PC MR is robust to parameter variations including encoding velocity, number of signal averages, voxel size, flip angle, slice thickness, and imaging acceleration (Greil et al., 2002; Correia de Verdier et al., 2024; Thunberg et al., 2003). Furthermore, PC acquisitions remain accurate despite practical acquisition challenges such as differing study sites, slice inclination, and breathing motion (Greil et al., 2002; Andersson et al., 2016; Summers et al., 2005). Because velocity is directly encoded in the moving blood, post-processing is straightforward and does not rely on complex modelling or assumptions that may differ between subjects who have normal or pathologic flow. By following well-established scanning norms such as minimizing repetition time and echo time, setting the encoding velocity to a value moderately above the fastest expected flow for a given clinical scenario, and selecting voxels sufficiently small to fit inside the vessel lumen, a robust PC protocols can be deployed in clinical environments across a wide age range (Greil et al., 2002; Correia de Verdier et al., 2024; Lagerstrand et al., 2020).

However, the clinical use of PC MR imaging to measure CBF is hampered when PC images from one or more vessels cannot quantify flow. Factors such as normal variation in vessel size, tortuosity of the ICAs and VAs, patient motion, and operator facility may contribute to an unsuccessful image acquisition. While automated techniques have been developed by researchers to overcome some of these challenges, they have not been implemented by vendors for use in standard clinical imaging environments (Liu et al., 2014). Our prior research in adults reported flow measurements in each vessel are sufficiently correlated with one another that imputation can compensate for measurements corrupted by anatomical variation, motion or obliquity, or improper placement (Shah et al., 2023). We previously reported that flow through any one vessel is highly correlated with CBF due to the left-right symmetry in arterial flows and the constant ratio between anterior and posterior flows, which lead to excellent imputation models in adults. This study extends the feasibility of imputation to a wider age range by examining these correlations in both children and adults. We developed new mathematical models using a combination of age and arterial blood flows to impute the CBF across the human lifespan.

## 2 Materials and methods

### 2.1 Patient demographics

This study includes 258 single-slice phase contrast images that were obtained from a total of 196 subjects (N = 108 children and N = 88 adults), across two studies. One study population comprised pediatric patients from a study approved by the Children’s Hospital Los Angeles Committee on Clinical Investigations (CCI 18–00493). Assent was obtained from the parents of patients (N = 67, all children) recruited between 2020 and 2023. These patients ranged from 0.4–11.9 years (μ = 2.7, σ = 1.6, 37M, 30F). This cohort included patients with seizures and epilepsy (N = 23), neoplasms (N = 14), developmental delay or regression (N = 7), cerebral palsy (N = 3), and other visit reasons (N = 20). All (N = 67) children were anesthetized with propofol (100%); some of these children also received dexmedetomidine (43%), sevoflurane (4%), or ketamine (1%). Their hematocrit levels were (μ = 35.9, σ = 3.2).

The other study population comprised unsedated patients and volunteers with ages ranging from adolescence through adulthood. This was a secondary analysis of existing data, which was originally approved by the Children’s Hospital Los Angeles Committee on Clinical Investigations (CCI 11–00083). Informed consent was obtained from 129 patients (N = 41 children and N = 88 adults) recruited between 2012 and 2017. These patients ranged from 9–61 years (μ = 23.5, σ = 9.7, 59M, 70F). This cohort included patients with sickle cell disease (N = 55), patients with other hemoglobinopathies (N = 32), and healthy control volunteers (N = 42). Their hematocrits levels were (μ = 32.9, σ = 7.2); some patients 33% of the adult cohort (N = 42) received chronic transfusions (Borzage et al., 2016). All subjects were free of known cerebral vessel steno-occlusive disease.

### 2.2 Image acquisition

The imaging methods are reported elsewhere in detail and summarized here (Borzage et al., 2016). We obtained all images for both groups with 3T Philips MRI systems (Ingenia, Ingenia CX, or Achieva) equipped with an eight-element head coil. We localized the vessels in the neck with a magnetic resonance angiogram, and then placed a PC MR imaging plane approximately 1 cm above the carotid bifurcation. In the pediatric population, the angiogram was collected in the sagittal plane; in the adult population, it was collected in the axial plane with inline reformatting into sagittal and coronal planes to facilitate orthogonal placement of the PC imaging plane. Because flow values are similar with and without cardiac gating, no gating was used (Nordmeyer et al., 2010). Imaging parameters for both protocols were similar (Table 1). Each pediatric patient yielded 1 to 3 PC MR images (36 scans yield 1 image each, 31 scans yielded 3 images each).

**TABLE 1:** Summary of relevant MR imaging parameters for pediatric and adult cohorts.

Exam	Pediatric	Adult
Echo Time (ms)	11	7.5
Repetition Time (ms)	140	12.3
Field of View (mm × mm)	100 × 100	260 × 260
Acquisition Matrix	112 × 107	204 × 201
Reconstruction Matrix	224 × 224	488 × 488
Slice Thickness (mm)	5	5
Bandwidth (Hz/pixel)	171	244
Velocity encoding gradient (cm/s)	60	200
Signal averages	36	10

### 2.3 Image processing

PC images were processed to quantify flow rate through each of the four major cerebral arteries. We performed all PC image analysis using ImageJ (National Institutes of Health, Bethesda, MD) and MATLAB (The MathWorks, Natick, MA) to quantify flow. The complex difference image was thresholded to identify moving voxels (defined as greater than the mean plus two standard deviations of stationary voxels sampled from a non-vascular region). Background phase was identified by fitting the phase differences of stationary voxels using a two-dimensional second-order polynomial and then removed. Vessel boundaries were automatically selected using a Canny edge-detector of the complex difference image, dilating the edge by a single voxel, creating a binary mask representing the inside of the vessel lumen, and then excluding any stationary voxels within the vessel mask. Blood flow in each artery was calculated by summing the blood velocities (cm/s) within the vessel multiplied by the voxel area (cm^2^). When the automatic edge detection failed (<5% of the time), the ICA or VA lumen boundaries were identified manually by an MR researcher (JCW) with 22 years of experience analyzing PC MR images. Images demonstrating successful acquisition and flow quantitation of the 4 vessels (2 ICAs, 2 VAs) underwent further processing and modelling.

### 2.4 Modeling cerebral blood flow as a function of vessels and age and other variables

We computed the mean and standard deviation for CBF, individual vessel flows, and anterior and posterior circulations. We evaluated all 4 blood flow ratios for left (L) and right (R) ICAs and VAs (LICA-CBF, RICA-CBF, LVA-CBF, RVA-CBF) as well as anterior-to-posterior and right-to-left flow ratios from both pediatric and adult volunteers with a two-way ANOVA. Post-hoc comparison of these groups was performed using a Wilcoxon Kruskal-Wallis test (Table 2).

**TABLE 2:** Wilcoxon Kruskal-Wallis test results demonstrate statistical differences in vessel-to-CBF ratios. Despite the statistical difference, the effect size was small. The median difference between the vessels ranged from −2.5% (pediatric RVA versus pediatric RVA) to 2.3% (pediatric RICA vs. pediatric LICA) (mean [μ] = 0.05% standard deviation [σ] = 2.2%). This small effect does not justify the use imputation models, for which 32 unique combinations of pediatric or adult vessels would be presented as separate imputation models. Note that this table omits comparison of ICAs versus VAs, which are clearly different.

Vessel 1	Vessel 2	p-value	Hodges-Lehmann pseudo-median	Lower confidence limit	Upper confidence limit
Pediatric RVA	Pediatric LVA	0.0004	−0.0246	−0.0383	−0.0112
Adult RVA	Pediatric LVA	0.0013	−0.0192	−0.0311	−0.0075
Adult LVA	Pediatric RVA	0.0219	−0.0137	−0.0261	−0.0022
Adult RICA	Pediatric LICA	0.0157	0.0168	0.0030	0.0300
Adult LICA	Pediatric LICA	0.0029	0.0210	0.0070	0.0352
Pediatric RICA	Pediatric LICA	0.0185	0.0230	0.0039	0.0408

We systematically excluded flow data from one or more vessels to simulate the effects of a sub-optimal image acquisition. We analyzed the 8 scenarios wherein combinations of 2, 1, or 0 ICAs or VAs could be analyzed from an image. We applied a standard least-squares model to estimate CBF as a function of (1) the remaining vessels able to be analyzed, and (2) an age-dependent equation. The age-dependent equation was a single parsimonious model of CBF as a function of age across the human lifespan. To create this equation, we evaluated age-based models (polynomial, power, logistic, and combinations thereof) using MATLAB (R2021b, Mathworks, Natick, MA), and compared their performance using root mean squared error. In scenarios where either 2 ICAs or 2 VAs were analyzed, we reduced model complexity by calculating the total anterior (sum of ICAs) or posterior (sum of VAs) flow to reduce degrees of freedom. The age-based model was extended to include the following additional parameters: sex; sex and hematocrit; and sex, hematocrit, and total brain volume.

### 2.5 Assessing cerebral blood flow model quality

The quality of each imputation model was assessed using root mean squared error, R-squared statistic, and intra-class correlation coefficient (ICC). The ICC (2, 1) is a two-way random, single measures absolute agreement between model 0 (gold standard, all vessels present) and age-based models 1–8 (Table 3), calculated using MATLAB. We also performed Bland-Altman analyses of all age-based models compared to known CBF (model 0). We calculated the biases as the mean difference between model 0 and each other model, and the 95% limits of agreement were defined as twice the standard deviation of the differences of individual measurements between model 0 and each other model.

**TABLE 3:** Models for computing CBF when vessels are missing. The models are independent of the lateral location of the imputed vessel(s), allowing us to omit models for the combinations of different left versus right vessels (Figure 3 **)**. Models 1–7 use an age-dependent model of CBF, denoted $\hat{CBF}\left(age\right)$ and defined as model 8, noted in Table 4 as model 8a; this is the most conservative of the vessel-independent CBF models. Overall, the models followed the anticipated pattern wherein missing ICAs contributed more error than missing VAs (e.g., model 3 versus 1). Age was useful in all models (p < 0.05), except model 1, for which it was not significant (p = 0.4454). Abbreviations cerebral blood flow (CBF), internal carotid artery (ICA), vertebral artery (VA), root mean square error (RMSE), intra-class correlation coefficient (ICC). An online calculator for these models is provided: https://brainflow.science/impute-cbf.

Number of Usable vessels		Model	Cerebral blood flow equation [mL/min]	p-value for term: $\hat{CBF}\left(age\right)$	RMSE	NormalizedRMSE	R-Squared	ICC(2,1)
ICA	VA							
2	2	0	Anterior + Posterior	NA	0	0​	1.00​	1.000​
	1	1	0.010 × $\hat{\text{CBF}}\left(\text{age}\right)$ +1.196 × Anterior+1.064 × VA	0.4454	46.34	0.063​	0.98​	0.990​
	0	2	0.045 × $\hat{\text{CBF}}\left(\text{age}\right)$ +1.376 × Anterior	0.0202	69.73	0.095​	0.95​	0.977
1	2	3	0.082 × $\hat{\text{CBF}}\left(\text{age}\right)$ +1.600 × ICA+1.192 × Posterior	<0.0001	52.14	0.071​	0.97​	0.987​
	1	4	0.111 × $\hat{\text{CBF}}\left(\text{age}\right)$ +2.003 × ICA+1.302 × VA	<0.0001	76.73	0.105​	0.94​	0.972​
	0	5	0.176 × $\hat{\text{CBF}}\left(\text{age}\right)$ +2.381 × ICA	<0.0001	100.49	0.137​	0.91​	0.951​
0	2	6	0.335 × $\hat{\text{CBF}}\left(\text{age}\right)$ +2.175 × Posterior	<0.0001	123.29	0.168​	0.86​	0.921​
	1	7	0.580 × $\hat{\text{CBF}}\left(\text{age}\right)$ +2.792 × VA	<0.0001	177.61	0.243​	0.70​	0.821​
	0	8	$\hat{\text{CBF}}\left(\text{age}\right)$ (See Table 4)	NA	236.95	0.324​	0.47​	0.638​

## 3 Results

In the pediatric volunteers, we report the following mean (μ) and standard deviation(σ) values for CBF (μ = 530.7, σ = 204.7), anterior flow (sum of ICAs, μ = 361.4, σ = 135.8), and posterior flow (sum of VAs, μ = 169.3, σ = 90.1). Flows in individual arteries were: LICA (μ = 176.4, σ = 75.3), RICA (μ = 184.9, σ = 73.3), LVA (μ = 90.4, σ = 51.2), and RVA (μ = 78.9, σ = 49.9).

**FIGURE 1:**
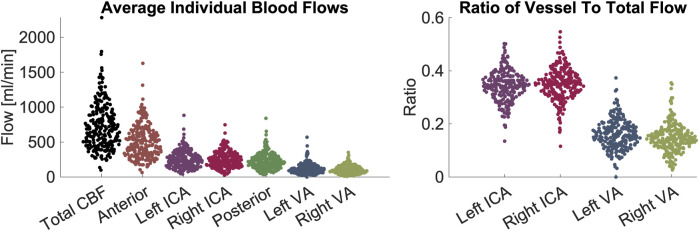
Demonstration of blood flow rates and ratios between vessels. Left panel: measurements of flow in units of mL/minute. From left to right the figures shows CBF contribution: total, anterior, left ICA, right ICA, posterior circulation, left VA, right VA. Right panel: ratios of flow in individual arteries versus CBF. From left to right the figure shows left ICA, right ICA, left VA, right VA. The figures demonstrate visually (1) the range of CBF (2), the contribution from individual arteries is predominantly from the anterior circulation, that flow through (3) ICAs and (4) VAs are symmetric on the left and right sides. Abbreviations cerebral blood flow (CBF), internal carotid artery (ICA), vertebral artery (VA).

**FIGURE 2:**
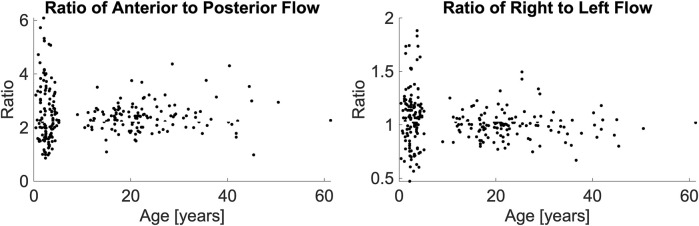
Anterior-to-posterior posterior ratio (left panel) and right-to-left ratio (right panel), presented as a function of their age. A Wilcoxon Kruskal-Wallace test demonstrated no difference between the pediatric and adult populations.

In the adult volunteers, we report the CBF (μ = 930.6, σ = 298.0), anterior flow (μ = 650.5, σ = 209.4), and posterior flow (μ = 280.0, σ = 105.9). Flows in individual arteries were: LICA (μ = 326.8, σ = 111.0), RICA (μ = 323.7, σ = 106.1), LVA (μ = 145.5, σ = 76.7), and RVA (μ = 134.5, σ = 53.2). All values are shown in Figure 1. Ratios of anterior-to-posterior flow and right-to-left flow are shown in Figure 2. Ranges of CBF are similar to previous studies usings color duplex sonography and 4D flow MRI (Schöning and Hartig, 1996; Wu et al., 2016).

The two-way ANOVA determined that differences in flow ratios were particular to vessel types (p < 0.0001) and the interaction of vessel type and age (p = 0.0147) but not age alone (p = 0.9312). We performed post hoc comparisons of these ratios with Wilcoxon Kruskal-Wallis tests. We found that for most ratios, both the age-based comparisons (e.g., ratios of pediatric LVA versus adult LVA) and the lateral comparisons (e.g., pediatric LVA-CBF versus pediatric RVA-CBF) to be statistically similar (p > 0.05, statistically different vessels reported in Table 2). As expected, all comparisons of ICA-CBF ratios and VA-CBF ratios (e.g., pediatric VA-CBF versus pediatric ICA-CBF) were different (p < 0.0001). However, the size of differences in ratios between individual vessels and CBF were minor; the maximum ratio difference (pediatric RICA-CBF versus pediatric LICA-CBF) was very small, 0.023. Due to this small effect size, we simplified the models using equivalence of vessel-to-CBF ratios across age groups and left and right symmetry of vessels.

We evaluated the difference in anterior-to-posterior and right-to-left flow ratios as a function hematocrit and age group. There was no association in a linear regression between hematocrit and either the anterior-to-posterior ratio (p = 0.0595) or right-to-left ratio (p = 0.8292). There was no association between age and either anterior-to-posterior flow ratio (p = 0.1928) or the right-to-left ratio (p = 0.3261).

**FIGURE 3:**
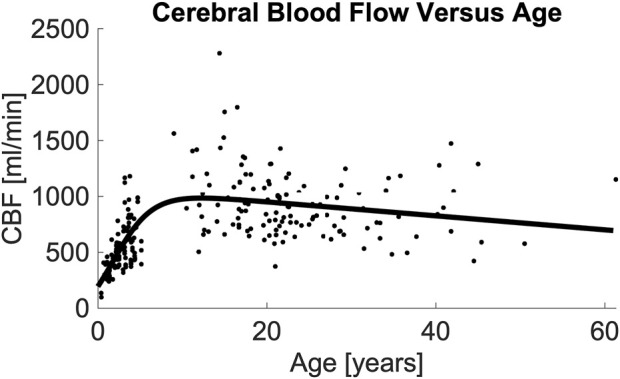
Image demonstrating total CBF vs. age and the resulting model. Regression analysis demonstrates that total CBF rises rapidly from birth through middle childhood followed by a slow decline from adolescence through adulthood. Regression line formula is noted in (Table 4, Model 8a). Abbreviations cerebral blood flow (CBF).

Table 3 demonstrates the performance of each imputation model as functions of the remaining vessels to be analyzed, and the age dependent equation. As anticipated, the error in imputation (RMSE) increased and the R-squared decreased from the model with the fewest to most missing vessels (model 1 to model 8). All models showed statistical significance (p < 0.0001); however the age term was not significant for model 1 (p = 0.4454), only marginally significant for model 2 (p = 0.0202), and significant for the remaining models (p < 0.0001). Table 4 demonstrates the vessel-free CBF models, using age, sex, hematocrit, and total brain volume. The age-logistic model to estimate age-related changes in CBF (Model 8a from Table 4, shown in Figure 3) was selected based on its superior performance over other tested models (Table 5). The same statistics were calculated for the original, age-independent imputation models (Table 6). Age-dependent models performed equivalently to the age-independent models in cases with 1 or 2 missing VAs, or 1 missing ICA with 1 or 2 VAs present (models 1, 2, 3, and 4), showing similar RMSE, normalized RMSE, ICC, and R-squared. In all other cases, the age-dependent models had lower RMSE and normalized RMSE, and higher R-squared correlations than the age-independent models.

**TABLE 4:** Vessel-free models of CBF. The presented models take parameters of age (years), sex, Hct, brainvol. Abbreviations cerebral blood flow (CBF, mL/min), root mean square error (RMSE), hematocrit (Hct, percentage), and total brain volume (brainvol, cubic centimeters).

Model	Cerebral blood flow equation [mL/min]	RMSE	Normalized RMSE	R-Squared
8a	$\hat{\text{CBF}}\left(\text{age}\right)=L/\left(1+b\times e^{\left(k\times \text{age}\right)}\right)+c\times \text{age}+d$ L = 1297, b = 2.132, c = −6.262, d = −220.5, k = −0.4247	236.95	0.324​	0.47​
8b	$\hat{\text{CBF}}\left(\text{age},\text{sex}\right)=L/\left(1+b\times e^{\left(k\times \text{age}\right)}\right)+c\times \text{age}+d$ If female: L = 777.8, b = 16.92, c = −8.456, d = 372.1, k = −0.5614 If male: L = 1101, b = 6.603, c = −2.742, d = 17.09, k = −0.7679	233.62	0.320	0.48
8c	$\hat{\text{CBF}}\left(\text{age},\text{sex},\text{Hct}\right)=0.804\times \hat{\text{CBF}}\left(\text{age},\text{sex}\right)-24.772\times Hct+966.895$	186.72	0.256	0.67
8d	$\hat{\text{CBF}}\left(\text{age},\text{sex},\text{brainvol},\text{Hct}\right)=0.5590\times \hat{\text{CBF}}\left(\text{age},\text{sex}\right)+0.5518\times brainvol-26.01\times Hct+675.8$	182.63	0.250	0.68

**TABLE 5:** Statistics comparing the performance of different models of cerebral blood flow as a function of age. The age-logistic model outperformed the other models, which failed to capture the rapid rise followed by slow decline of cerebral blood flow with age. Models are named by their corresponding fit type in MATLAB, while the age-logistic formula is presented in Table 4, Model 8a.

Fit type	Number of Coefficients	R-Squared	RMSE
Age-logistic	5	0.47	238.81
Power 2nd order	3	0.41	250.24
Power 1st order	2	0.35	262.66
Polynomial 2nd order	3	0.33	266.26
Polynomial 1st order	2	0.22	287.86
Exponential 1st order	2	0.18	293.88

**TABLE 6:** Statistics for performance of age-independent models on adult and pediatric data. Models 1–7 use an age-independent model of CBF with the same configuration of missing vessels as Table 1; model 8 is defined as the population mean from the age-independent study. Performance of these models was equal to or worse than their corresponding age-dependent models. Abbreviations cerebral blood flow (CBF), internal carotid artery (ICA), vertebral artery (VA), root mean square error (RMSE), intra-class correlation coefficient (ICC).

Number of Usable vessels		Old model	Cerebral blood flow equation [mL/min]	RMSE	NormalizedRMSE	R-Squared	ICC(2,1)
ICA	VA						
2	2	0	Anterior + Posterior	0	0​	1.00​	1.000​
	1	1	1.226 × Anterior+0.933 × VA	45.91	0.063	0.98	0.986
	0	2	1.426 × Anterior	69.74	0.096	0.95	0.977
1	2	3	1.886 × ICA+1.145 × Posterior	56.07	0.077	0.97	0.985
	1	4	2.419 × ICA+1.983 × VA	80.76	0.110	0.94	0.970
	0	5	2.841 × ICA	111.42	0.154	0.89	0.941
0	2	6	3.219 × Posterior	157.42	0.217	0.81	0.896
	1	7	5.816 × VA	254.03	0.380	0.61	0.752
	0	8	933.656	324.65	0.443	NA	0.000

**FIGURE 4:**
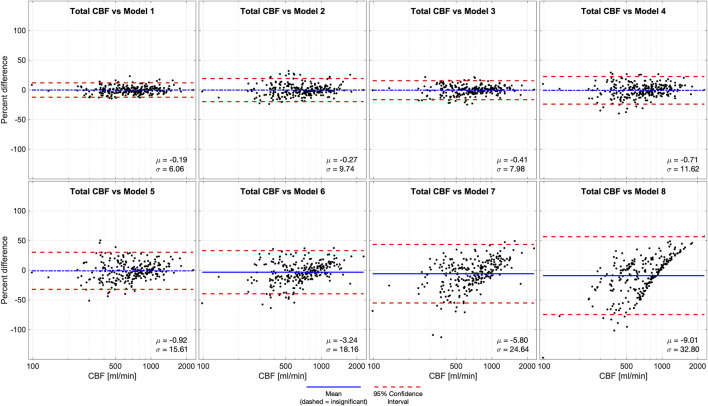
Bland-Altman analysis of total CBF quantified by phase contrast (PC) in 4 vessels (model 0) versus various models of 1 or more missing vessels. Blue line demonstrates bias, dashed blue line demonstrates insignificant (p > 0.05) bias, and red dash lines demonstrate 95% confidence intervals. These results suggest that it is important to successfully capture at least 1 ICA or 2 VAs to impute CBF measurements. Age-dependent model 4 was unbiased, improving performance compared the previously published age-independent model 4. Abbreviations cerebral blood flow (CBF).

We performed Bland-Altman analyses of models 1-8 compared to ground truth (model 0) (Figure 4). Models 1-5 were statistically unbiased (p > 0.05), while model 6 had a small yet significant bias (μ = −3.24%, p = 0.0045). Models 1-4 had narrow limits of agreement (σ = 6.06–11.62%) while models 5-6 and had wider limits of agreement (σ = 15.61–18.16%). Model 7, which was derived from a measurement in 1 VA, it performed exceptionally poorly (μ = −5.80%, σ = 24.64%, p = 0.0002), similar to model 8a (μ = −9.01%, σ = 32.80%, p < 0.0001) where CBF is estimated based on age alone.

## 4 Discussion

Cerebral blood flow varies considerably across the lifespan, especially across infancy and childhood. PC-based CBF measurements provide a robust and fast complement to both regional MR perfusion techniques, (arterial spin labelling and dynamic susceptibility contrast), bedside techniques (3D Doppler ultrasound), and radiologic approaches such as PET and SPECT (Clement et al., 2022; Shen et al., 2023, Catafau et al., 2001, Frackowiak et al., 1980). Two-dimensional PC methods require minimal scan time, complexity, or post-processing requirements, making them easier to deploy than more advanced 4D PC. We therefore sought to extend our previous imputation models by incorporating age-dependence and build models applicable across the human lifespan. The new age-based models meet or exceed the performance of the previously published age-independent models in all cases. We further evaluated other variables (hematocrit, sex, and brain volume) and present models that use these parameters to improve imputation. However, the presence of at least one well-resolved ICA remains crucial for performing high-accuracy imputation. In fact, when two ICAs and one VA were present, the age dependence in the imputation (model 1) was found to be statistically insignificant. Similarly, when both ICAs are present (model 2), the contribution of the age-dependent term was marginal. For the remaining models, the inclusion of age dependence was statistically significant and improved the quality of the models. Taken together, this suggests that imputation using age does not harm any of the models and may help improve accuracy, particularly when ICAs are not measured. The relatively modest effect of age in the modelling is attributable to the fact that anterior-to-posterior and right-to-left flow ratios do not change substantially with age, although total CBF does change with age, and these ratios have relatively high intra-subject variability unexplained by age.

Bland-Altman analysis of CBF versus imputed CBF demonstrated a similar trend. Models 1–5 were statistically unbiased and show similar 95% confidence intervals to the age-independent models. Despite the improved performance of all models, the Bland-Altman analysis still showed a significant bias for age-dependent models 6–8 with wide 95% confidence intervals. This confirms that failing to measure at least one ICA causes significant degradation of imputation performance. We included models 6 and 7 because they were valid correlates of CBF, and model 8a (CBF as a function of age) for completeness, but we recommend against using them for CBF imputation.

We anticipated and reported differences in vessel-to-CBF ratios for children and adults. However, these statistically significant differences were subtle, indicating only modest redistribution of flow patterns from childhood (when it is slightly more posterior) to adulthood (more anterior). This contrasted with our anticipation of a more profound change across development. The age-dependence of CBF follows a generally similar trend to total brain volume with age, peaking in late adolescence to early adulthood and slowly declining over the remainder of the human lifespan (Bethlehem et al., 2022; Borzage et al., 2014).

Although our models were intended for data imputation, they may also provide value in the case of the clinical setting. An unsuccessful acquisition would normally be repeated by a technician, however, occasionally an error is missed until after the patient is no longer being scanned, or repeating a scan is not possible because a patient is unstable. In these admittedly rare cases, our improved imputation provides more accurate results. A more likely application in a clinical setting is using our imputation after a successful acquisition. When all four vessels are acquired, our models can be used to evaluate potentially abnormal flow patterns due to intracranial arterial stenosis diminishing flow in one or more vessels (Kim et al., 2022; Craig et al., 1982). This evaluation comprises: calculating ground truth CBF (model 0), omitting one or more vessels and imputing CBF (model 1, 3), then comparing ground truth versus imputed CBF. Larger CBF discrepancies indicate flows that do not follow our established patterns. An alternative application in a clinical setting is detecting abnormal flow as a function of age, which may indicate impaired brain development.

## 5 Limitations

A key assumption of these models is that the cerebrovasculature is anatomically complete. The participants included in the modelling do not have steno-occlusive disease, and our imputation models are suitable for studying development and aging but will likely fail in subjects with dysfunctional vasculature, such as diminished flow through one or more vessels. Techniques such as arterial spin labelling will be more appropriate for quantifying blood flow and characterizing steno-occlusions in persons with vascular dysfunction. Brain volumes were not obtained in the analysis so rCBF [mL/min/100 g] cannot be reported; the process for brain volume normalization is presented in Supplementary Material for interested readers. The CBF imputation models presented assume that the anterior-to-posterior and right-to-left flow ratios are constant. Work is ongoing to determine whether these ratios, especially anterior-to-posterior flow, remain constant in the presence of anesthetics; application of these models will demonstrate increased error if the anterior-to-posterior ratio is modified by diseases or interventions. Furthermore, model 8a demonstrates that a smooth function with respect to age does not capture the high variation in CBF between two individuals. CBF is influenced by a wide variety of intrinsic and extrinsic factors, including key covariates such as sex and hematocrit (Borzage et al., 2016; Bush et al., 2016; Clement et al., 2018). The more complicated models presented (8b, 8c, 8d, Table 4) moderately improve performance by including hematocrit, sex, and total brain volume, but this limits their applicability to datasets which include these details. Our data were acquired with different protocols as a part of two separate projects with MR imaging for different indications. The pathologies and use of anesthesia in the younger group differed considerably from those in the older group. Therefore, we cannot exclude the possibility of important covariates or confounders that we cannot ascertain with this data. Average flow rates were assessed by our pipeline but peak flow rate data was not recorded. We also lack external data validation from other institutions. The full range of our data is 0.4–61.3 years, however most (95%) of our data spans 0.75–4.85 and 11.20–45.10 years, thus we recommend caution when attempting to extend this model into ages <0.75, 4.85–11.20, or >45.1 years, which represent an opportunity for future work. However, the effects of the limited data on the age-based CBF model are limited by the parsimonious nature of the equation. We also found only subtly different patterns of anterior and posterior flow in our two groups, which we omitted when modeling the ratio between CBF and vessel (Bethlehem et al., 2022). These limitations present opportunities for future research, particularly in studies that recruits volunteers with similar characteristics across the lifespan.

## 6 Conclusion

Phase contrast MR enables effective, robust estimation of CBF in both pediatric and adult populations. Our findings suggest that employing age-dependent imputation models, based on at least one ICA flow, offers a single, parsimonious approach to estimating CBF across the human lifespan when one or more vessels cannot be directly measured. This reduction in number of acquisitions and total scan time required for CBF estimation reduces the need for on-scanner quality control measures and mitigates attendant challenges.

PC MR imaging, combined with our methods for addressing missing data, provides valuable insights into CBF and the metabolic sustenance of the brain. This approach can complement other MR imaging techniques suited for assessing lung and heart efficiency in oxygenation and blood delivery, or for mapping the regional distribution of blood and oxygen consumption in the brain. Thus, PC MR fulfills a critical role in understanding brain physiology. Our imputation models demonstrate a significant advancement over previous approaches in describing vascular physiology. Our equations simplify CBF measurement across diverse ages groups and offers a solution to real-world acquisition challenges.

## Data Availability

The raw data supporting the conclusions of this article will be made available by the authors, without undue reservation.
