# Supplementary material for: Imputation models and error analysis for phase contrast MR cerebral blood flow measurements in heterogeneous pediatric and adult populations
Source: Front Physiol. 2025 Jun 11;16:1527093. doi: 10.3389/fphys.2025.1527093 (PMC12187604; doi:10.3389/fphys.2025.1527093)
Supplement: Supplementary file 1 [file Supplementaryfile1.docx]

Supplementary Material

Imputation Models and Error Analysis for Phase Contrast MR Cerebral Blood Flow Measurements Throughout the Human Lifespan

Eamon K. Doyle, Isabel Torres, Joseph Liu, Abhishek Karnwal, Sudarshan Ranganathan, Bradley J. De Souza, Payal Shah, Bradley S. Peterson, John C. Wood, Matthew Thomas Borzage^*^

*** Correspondence:**Eamon K. Doyle
[edoyle@chla.usc.edu](mailto:edoyle@chla.usc.edu)

Matthew T. Borzage
[Borzage@usc.edu](mailto:Borzage@usc.edu)

# Supplementary Normalization of Cerebral Blood Flow by Brain Volume or Weight

Models 1-8 in the main text of our manuscript are suitable for imputing one or more missing arteries, each model returns a cerebral blood flow (CBF) in units of ml/min. Our models are based on ratios of flows in which each parameter is a dimensionless scaling factor. Thus, the models only require measurement of the arterial flow(s) in the PC image, computation of model 8 using age as the input, and then apply our equations to estimate total CBF (ml/min).

Total CBF is often normalized to brain weight and reported in units of ml/100g/min (1). This is approach is both very useful and very common. Normalized CBF (nCBF) usefully adjusts for the size of the brain to better characterize the perfusion demand; nCBF is common because several CBF measurements are based on totaling up regional perfusion in imaging voxels. Thus, nCBF values are frequently demonstrated in studies based on single photon emission computed tomography, positron emission tomography, dynamic susceptibility contrast MR imaging, or arterial spin labeling MR imaging (2).

We excluded a consideration of brain weight or volume measurements from our basic models for several reasons. First, brain weight was not required because our methods involve dimensionless ratios of flow in large arteries. As demonstrated in the main text, our ratios **(Figure 1)** were generally stable across the lifespan **(Figure 2),** despite the considerable change in brain size across the lifespan. Second, measuring brain size involves some degree of measurement error (3). Introducing the brain size into our equations would propagate that error into our models. Third, our models can be applied without acquiring brain images for volume/weight estimation, providing immediate utility to readers lacking access to brain size while not precluding application in environments where brain size is available. If the reader does have access to brain size, then normalizing the CBF by the brain size is trivial. Readers lacking access to brain size can review appropriate literature, find suitable brain size measurements or models, and use the instructions below to convert to normalized CBF. However, the reader must also evaluate the contribution of their brain size measurements to the errors we present in the main text **(Table 1)**.

Once a reader has an accurate brain size, converting models 0-8 in our main text from units of ml/min to units of ml/100g/min is straightforward. A reader takes the CBF model results, divides by the brain weight, and then multiplies by 100 **(SM Equation 1)**. If the reader has brain volume measurements (e.g. from imaging), then they convert volume to weight by multiplying by a density factor **(SM Equation 2)**. However, the processes for obtaining brain weight from autopsy, brain volumes from medical images, or brain density are all beyond the scope of our methods.

$normalized CBF=\mathrm{CBF}/{(brain weight)} \times100$ SM Equation 1

$normalized CBF=\mathrm{CBF}/{(brain volume \times brain density)} \times100$ SM Equation 2

Thus, should the reader choose to do so, they can combine model 8 from the main text and **SM Equations 1, 4** to yield Model 8B. This new (normalized) model can then be combined with models 1-7 to provide normalized CBF values:

$\hat{normalized CBF}\left( \mathrm{age} \right)=\frac{L/\left( 1+b\times e^{\left( k \times age \right)} \right)+c \times age+d}{\left[ brain weight \right]} \times100$ Model 8B

Assuming the reader does not have ready access to brain size information, we present one published analytical estimate of brain weight as a function of age and sex (4).

$\frac{d\left[ brain weight raw \right]}{d\left[ age \right]}=m \times\left[ brain weight raw \right] \times\frac{\left( n-\left[ brain weight raw \right] \right)}{\left( n-\left[ brain weight raw \right]+o \times\left[ brain weight raw \right] \right)}$ SM Equation 3

$[brain weight]=[brain weight raw] \times\left( 1+p \times\left[ {log}_{10}(age-r) \right]+q \times\left[ {log}_{10}(age-r) \right]^{2} \right)$ SM Equation 4

Using this estimate requires solving an ordinary differential equation (SM Equation 3) using an initial condition (brain weight = 1 gram) and the parameters which depend on sex: male (m=46, n=746, o=6.83, p=1.37, q=-0.502, r=1.75), female (m= 45.1, n=852, o=6.69, p=0.821, q=-0.314, r=1.75). However, this model does not present a root mean squared error, and thus understanding the degree of error propagation in the normalized CBF values is not straightforward. If these normalizations are used, then they should be used with considerable caution and understanding of the limitation of modeling brain size.

# Supplementary References

1. Clement P, Petr J, Dijsselhof MBJ, Padrela B, Pasternak M, Dolui S, et al. A Beginner’s Guide to Arterial Spin Labeling (ASL) Image Processing. Front Radiol [Internet]. 2022 Jun 14 [cited 2024 Aug 23];2. Available from: https://www.frontiersin.org/journals/radiology/articles/10.3389/fradi.2022.929533/full

2. Zhao MY, Tong E, Duarte Armindo R, Woodward A, Yeom KW, Moseley ME, et al. Measuring Quantitative Cerebral Blood Flow in Healthy Children: A Systematic Review of Neuroimaging Techniques. J Magn Reson Imaging. 2024 Jan;59(1):70–81.

3. van Nederpelt DR, Amiri H, Brouwer I, Noteboom S, Mokkink LB, Barkhof F, et al. Reliability of brain atrophy measurements in multiple sclerosis using MRI: an assessment of six freely available software packages for cross-sectional analyses. Neuroradiology. 2023 Oct 1;65(10):1459–72.

4. Borzage M, Blüml S, Seri I. Equations to describe brain size across the continuum of human lifespan. Brain Struct Funct. 2014 Jan;219(1):141–50.
